# Improving Health Monitoring With Adaptive Data Movement in Fog Computing

**DOI:** 10.3389/frobt.2020.00096

**Published:** 2020-09-15

**Authors:** Cinzia Cappiello, Giovanni Meroni, Barbara Pernici, Pierluigi Plebani, Mattia Salnitri, Monica Vitali, Diana Trojaniello, Ilio Catallo, Alberto Sanna

**Affiliations:** ^1^Dip. Elettronica, Informazione e Bioingegneria, Politecnico di Milano, Milan, Italy; ^2^Center for Advanced Technology for Health and Wellbeing, IRCCS San Raffaele Hospital, Milan, Italy

**Keywords:** data utility, fog computing, data movement, data analytics, data quality, quality of service

## Abstract

Pervasive sensing is increasing our ability to monitor the status of patients not only when they are hospitalized but also during home recovery. As a result, lots of data are collected and are available for multiple purposes. If operations can take advantage of timely and detailed data, the huge amount of data collected can also be useful for analytics. However, these data may be unusable for two reasons: data quality and performance problems. First, if the quality of the collected values is low, the processing activities could produce insignificant results. Second, if the system does not guarantee adequate performance, the results may not be delivered at the right time. The goal of this document is to propose a data utility model that considers the impact of the quality of the data sources (e.g., collected data, biographical data, and clinical history) on the expected results and allows for improvement of the performance through utility-driven data management in a Fog environment. Regarding data quality, our approach aims to consider it as a context-dependent problem: a given dataset can be considered useful for one application and inadequate for another application. For this reason, we suggest a context-dependent quality assessment considering dimensions such as accuracy, completeness, consistency, and timeliness, and we argue that different applications have different quality requirements to consider. The management of data in Fog computing also requires particular attention to quality of service requirements. For this reason, we include QoS aspects in the data utility model, such as availability, response time, and latency. Based on the proposed data utility model, we present an approach based on a goal model capable of identifying when one or more dimensions of quality of service or data quality are violated and of suggesting which is the best action to be taken to address this violation. The proposed approach is evaluated with a real and appropriately anonymized dataset, obtained as part of the experimental procedure of a research project in which a device with a set of sensors (inertial, temperature, humidity, and light sensors) is used to collect motion and environmental data associated with the daily physical activities of healthy young volunteers.

## 1. Introduction

The huge potential of the Internet of Things (IoT) paradigm has been immediately understood and applied in many domains (Ahmed et al., 2016) to provide advanced sensing layers, enabling solutions for personal needs (e.g., monitoring daily activities) (Alaa et al., 2017) as well as services for entire communities (e.g., smart cities) (Zanella et al., 2014). In the healthcare domain, IoT has also been adopted to similarly improve current processes and to provide new services to patients. However, extensive use of IoT data with continuous data flows from monitored patients can pose several challenges in developing effective and performing systems. For instance, the continuous and real-time monitoring of body parameters has so far been dedicated only to critically ill patients admitted to an intensive care unit. In any other case, to reduce the amount of transmitted data, either the patients are monitored at a lower rate (e.g., with a daily or weekly visit at the hospital) or, even if the sensors are able to constantly monitor patients, the monitoring data are collected from time to time (for example, the data are sent to the hospital every morning). In addition, the development of a system becomes more complex if we consider that the monitoring of a patient could require different types of sensors from different manufacturers, thus requiring a significant effort for integrating them and their underlying processes (Vitali and Pernici, 2016).

Although the IoT paradigm is addressing most of these challenges by considering the contribution of different communities, such as device manufacturers, network managers, internet-based solution providers, and semantic web researchers (Atzori et al., 2010), additional effort is required to create a more fruitful collaboration between the sensing layer and the application layer. While the former is focused on solving problems related to the observation and measurement of physical phenomena and the digital representation of such measurements, the latter is in charge of analyzing the sensed data to provide information and knowledge. Focusing on the deployment of this type of systems, the sensing layer is usually located on the edge, while the application layer is located on the cloud because it provides a more scalable and reliable infrastructure. On the other hand, there are situations in which the analysis (or a part of it) cannot be executed on the cloud. For example, data could not be moved from the premises for privacy reasons. Also, in the case of big datasets, moving all the data to the cloud for processing may take too long. Therefore, a more articulated deployment of the application layer—involving both the edge and the cloud—is required. In this context, Fog Computing (IEEE, 2018) has been introduced as a paradigm for creating applications able to exploit both the cloud and edge computational power as well as the devices in between to create a continuum between the two sides. This is particularly important in healthcare applications since data related to users are sensitive by definition. Their analysis and storage must therefore comply with current regulations, such as the General Data Protection Regulation (GDPR) (Ducato, 2016). At the same time, health monitoring solutions should be flexible with respect to the type of users. For instance, there are situations (e.g., emergencies) where the ability to provide rapid analysis is more important than having a 100% accurate result that could take an unacceptable period of time to be computed. Conversely, when the data are collected for diagnostic reasons, data accuracy is more important than their freshness.

The aim of this work is to present how the principles of the Fog Computing paradigm can be adopted to improve health monitoring with the aim of providing information at the right time, in the right place, and with the right quality and format for the user (D'Andria et al., 2015). For this reason, the proposed framework is based on two main elements:

- The *data utility* concept (Cappiello et al., 2017), which provides a quantitative evaluation of the relevance of the data obtained as the combination of two factors: (i) data quality, related to the fitness for use, which includes dimensions like accuracy, volume, and timeliness, and (ii) Quality of Service (QoS), related to the performance of the data delivery, which depends on the mutual location of where the data are stored and where they are used. Given a data source, not all the users have the same utility requirements of that data source. Also, the network can have different impacts on the user experience when accessing the data source. Given these assumptions, the data utility is assessed in two steps. First, a Potential Data Utility (PDU) is calculated to evaluate the data utility of a data source independently of a specific user. Second, when the user is known, the PDU is refined to obtain the actual data utility specific to the user's requirements.
- A *goal-based model* (Plebani et al., 2018), which is adopted to specify the requirements for the application layer with respect to the data utility. Compared with the typical goal-based models adopted in requirements engineering, the solution proposed in this paper also includes an additional *treatment layer*, following the methodology proposed in Vitali et al. (2015), which includes the adaptation actions available and the impact of the enactment of these actions on meeting the requirements.

Consequently, the combination of these two components offers to the Fog environment a tool to select the best strategy for copying or moving data between the different storage units whilst also considering the possible transformations required and the impact of the network. Given the requirements specified for the application, our framework reacts to their violations by selecting the best adaptation action. Since we are dealing with a dynamic environment, the best strategy as well as the impact of an action over the application requirements can change over time. The framework also takes this aspect into account.

The proposed approach was evaluated in the healthcare scenario, where the interests for data could vary for different users. For example, for a clinical expert monitoring a particular patient, the data must be detailed and promptly available. Conversely, when a clinician is performing data analysis for research purposes, coarse-grained data—requiring less network bandwidth—may be sufficient.

The rest of the paper is organized as follows. section 2 introduces the main characteristics of Fog Computing to the reader, while section 3 discusses the motivating example used throughout the paper. section 4 focuses on the data utility concept, and section 5 identifies the adaptation actions that could affect the data management. section 6 details the characteristics of the enriched goal-based model used to select the best adaptation actions, whose evaluation is illustrated in section 7. Finally, section 8 discusses related work on the data movement in Fog environments, and section 9 concludes the work outlining possible future work.

## 2. Fog Computing

**Figure 1:**
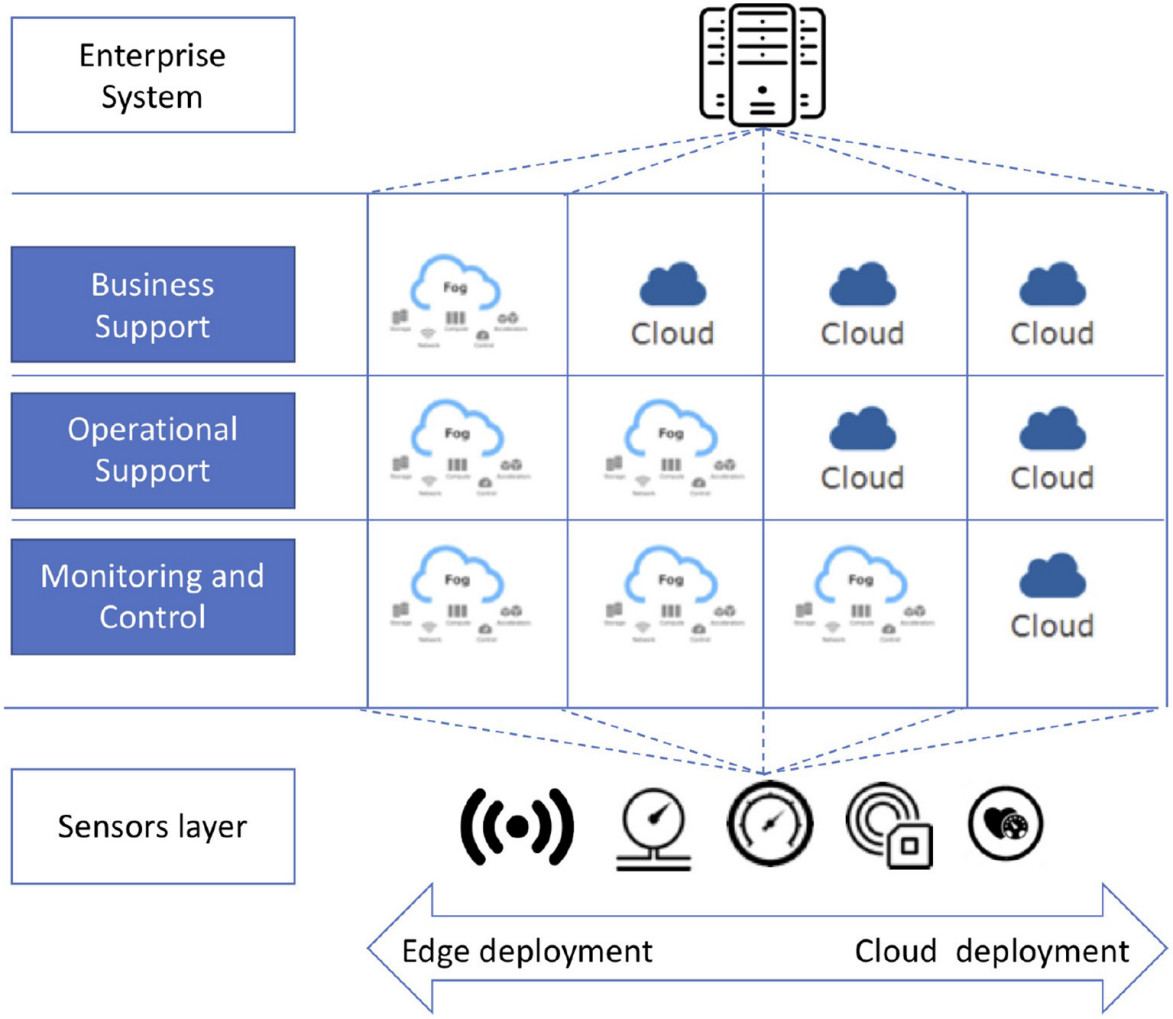
Possible deployment models in Fog Computing (inspired by IEEE, 2018).

Fog computing is emerging as a paradigm for the design, development, implementation, and maintenance of applications that are not necessarily distributed in the same environment—either cloud or edge—but which could also be allocated to resources in between (e.g., cloudlets) (IEEE, 2018). This paradigm has been mainly conceived to have in mind IoT (Internet of Things)-based applications, which can be organized around four main layers: (i) the sensors and actuators, where the data are generated or actions to the environment have effects; (ii) the Monitoring and Control, where the state of the application is monitored and controlled; (iii) the Operational Support, where a deeper analysis of the produced data is performed; and (iv) the Business Support, where data about different environments are collected and analyzed as a whole. In particular, when referring to Fog computing, different deployment models can be adopted (see Figure 1) with the aim of exploiting not only the resources available on the cloud but also those on the edge and in the infrastructure layers connecting these two environments. Based on this configuration, when the scalability of a solution is a key issue, a cloud deployment is preferable, as it provides a virtually unlimited amount of resources. Conversely, when latency must be reduced as much as possible and/or privacy constraints require that data should not leave the location where they are generated, a deployment on the resources running on the edge is preferred.

On this basis, the Fog computing paradigm considers a data flow that mainly moves data from the edge, where the data are generated, to the cloud, where they are processed. However, devices on the edge are getting more and more powerful in terms of computational and storage resources. According to this, Fog computing takes advantage of these resources by distributing the computation among the layers. In each layer, data are processed and analyzed to provide a synthesis for the layer above. In this way, the amount of data that should be moved decreases layer by layer. Moreover, the resulting data aggregation enables a mitigation of the data privacy related issues.

Although there is consensus around this view of Fog computing, such a paradigm must be more than creating a data center in the box, i.e., Cloudlets (Satyanarayanan et al., 2009), to bring the cloud closer to data producers. Instead, Fog Computing must be seen as a “resource layer that fits between the edge devices and the cloud data centers, with features that may resemble either” (Varshney and Simmhan, 2017). In particular, as discussed in Bermbach et al. (2018), the principles of Service Oriented Computing can be valuable also for Fog-based solutions to create a set of services able to simplify the data management in a Fog infrastructure in terms of new abstraction models able to hide the details of smart devices living on the edge of the network that could be very heterogeneous. Moreover, the effort in Fog computing should also be focused on simplifying resource management while considering both edge and cloud resources.

In this direction, the DITAS project^1^ is focusing on improving data-intensive applications by exploiting the peculiarities of Fog infrastructures, starting from the observation that most of the data, especially in IoT scenarios, are generated on the edge and are usually moved to the cloud to perform the required analyzes. While doing so can improve the performance of such data analysis due to the capacity and scalability of cloud-based technologies, there are situations in which this approach is not convenient or even impossible. For instance, when the amount of data to be analyzed is significant, the effect of the network may be considerable^2^. Furthermore, for privacy reasons, the owner of the data may not allow the movement of data outside of the boundaries of the organization unless they are anonymized and, in some cases, such an anonymization could hamper the analysis. On the other hand, limiting the computation to the resources on the edge could reduce the performance as the amount of resources, and their capacities are generally limited.

The depicted scenario is perfectly suited to the e-health domain where the data are heterogeneous (e.g., structured and unstructured data, images, and videos) and produced by heterogeneous devices; privacy is another a key issue, and the analysis of these data is complex, and, in some cases (e.g., during emergencies) it must performed quickly. Focusing on a single data analysis process, the adoption of the Fog computing paradigm can be helpful. Indeed, the computation can be organized hierarchically on the devices from the edge to the cloud, each of them specialized on some operations. Conversely, this approach cannot be so helpful in case there are many operators aiming to analyze in different ways the same dataset. In this case, there is a risk of having several deployments, each of them attempting to reach a local optimum, without any coordination in managing the common resources, like the computational power and the network bandwidth.

**Figure 2:**
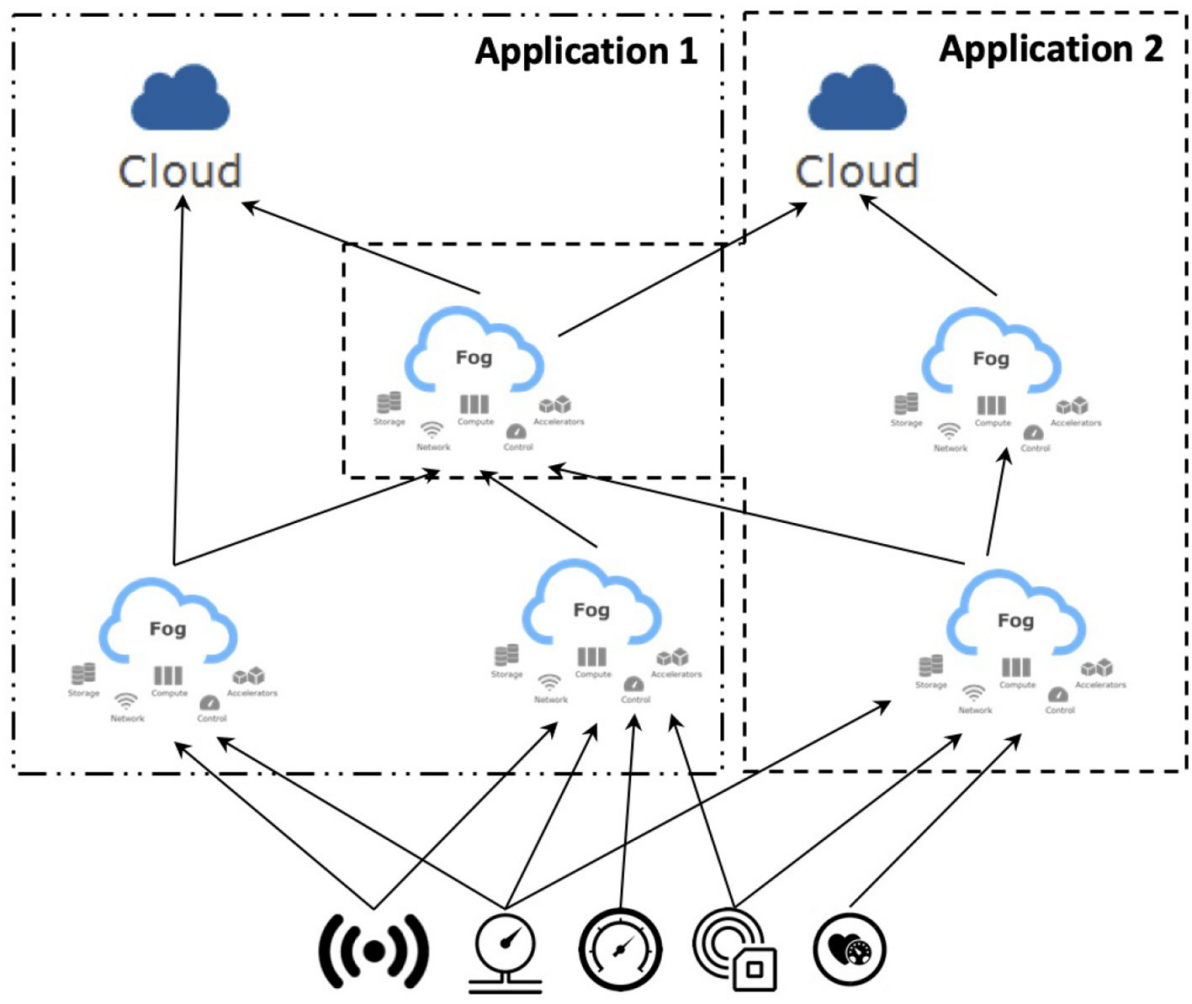
Fog deployment in DITAS.

Focusing on the optimization of the data movement, it is fundamental to properly manage the information logistics (Michelberger et al., 2013), i.e., the delivery of information at the right time, in the right place, and with the right quality and format to the user (D'Andria et al., 2015). As a consequence, user requirements can be defined in terms of functional aspects, i.e., contents, and non-functional ones, i.e., time, location, representation, and quality (Plebani et al., 2017). To this aim, it is crucial to define a proper set of strategies to enable data management involving the resources in the Fog to enforce a given data utility (Cappiello et al., 2017). As shown in Figure 2, the DITAS project investigates the possibility to manage the deployment of applications which are based on the same data sources. In this way, the resources in the Fog for a given application can be organized according to a hierarchical topology. At the same time, when the same processing is required for different applications, the deployment approach could either go for a duplication of the computation nodes or allow a node to be shared among different applications. Regardless of the deployment strategy, which is out of the scope of this article, proper data management among the different nodes involved in all the considered applications is required due to the motivations discussed above. For instance, the data coming from the sensors can be collected in the data storage of a gateway close to the sensor layer. At the same time, different replicas of these data have to be put in place to serve some of the fog nodes. Since the type of computation performed on these nodes can vary, the frequency and type of data to be transmitted to these nodes can also vary.

## 3. Motivating Example

**Figure 3:**
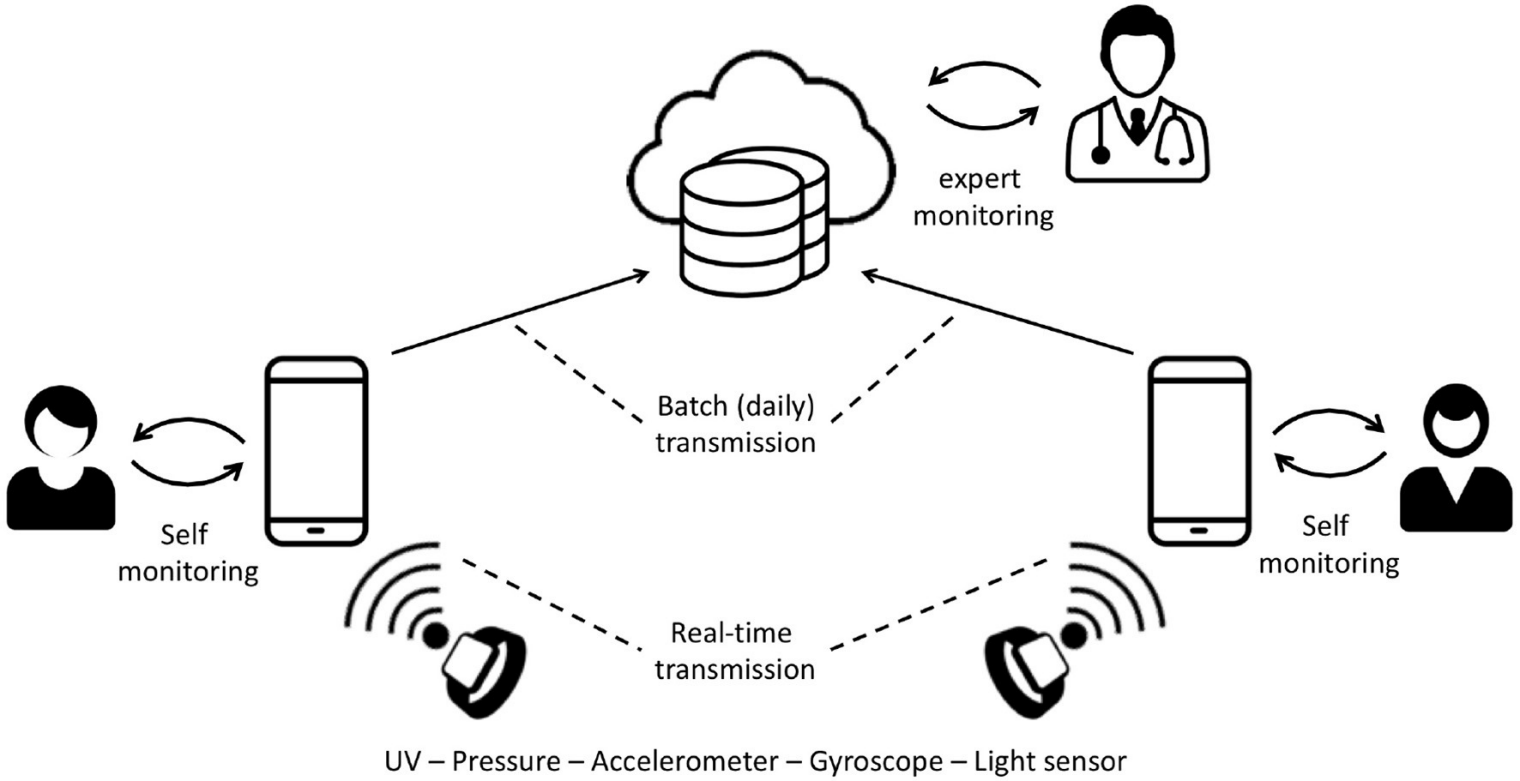
Data flow in the motivating scenario.

To properly introduce the approach proposed in this paper, we here describe a reference example, related to the usage of wearable devices as a means of facilitating patient monitoring. Indeed, in recent years, thanks to technology advances in the field of miniaturized sensors, various innovative wearable technologies have been developed. The introduction of such technologies in daily routines has raised great interest in new means of data collection in healthcare research and clinical contexts. Multiple applications for wearable devices have been identified in different areas of prevention, therapy, and well-being, ranging from the collection of relevant clinical data such as heart rate variability (HRV) to daily monitoring of physical activity. Furthermore, the possibility of collecting environmental parameters that could affect the subject's well-being through wearable devices is considered of great interest. In a recent H2020 project (I-SEE^3^), a new wearable device has been proposed that integrates a series of sensors, including UV, pressure, accelerometer, gyroscope, and light sensors. As depicted in Figure 3, in the scenario considered in this paper, the data collected by the wearable device are sent to a mobile application via Bluetooth. Part of the data processing is running on the wearable device and part on the mobile application. The data collected by the mobile application are daily (automatically) sent to the cloud. One of the main characteristics of the system is the presence of the UV sensor. Indeed, prolonged human exposure to solar UV radiation can have acute and chronic health effects on the skin, eyes, and immune system. In the long run, UV radiation could also induce an inflammatory eye reaction. During outdoor activities, it is therefore important to be protected from UV rays to avoid their harmful effects especially for children, athletes, and individuals with the pre-maculopatia diagnosis. The availability of the UV sensor, correctly mounted on the wearable device, therefore allows the system to collect data on exposure to UV light during the day, consequently allowing for two interesting and clinically relevant applications:

- Self-monitoring: the UV sensor measures the amount of UV-A and UV-B and informs the user, through the mobile application, about the current UV exposure and related risk (on the basis of their risk profile, properly computed thanks to the user information collected through the mobile app). In case of overexposure, the mobile application alerts the user and proposes a solution in order to meet the compliance parameters. Since in the mobile application data are automatically saved in the cloud, the user could also verify, through a diary, their UV exposure condition over the last months. In addition, further useful insights for the user come from the combined analysis of the large amount of data collected by other users who experiment similar exposure conditions, e.g., the user can check their condition with respect to other people with similar risk profiles (e.g., age, sex, and photo-type) in the same geographical area. The user can also define a pool of other users (e.g., family members) who will be able to access their data.
- Expert-monitoring: the data collected and saved in the cloud could also be queried by clinical experts (e.g., dermatologists). Through a specific Web application, clinicians can remotely monitor patients and, when a risky condition occurs, invite them for a clinical visit. In this application, the possibility to access data of a large number of users allows the experts to obtain insights on different patients populations, based on the age, the sex, and other relevant features.

Both applications require the analysis of the collected raw data in order to extract meaningful information (i.e., UV intensity, time spent under UV, and time over risk thresholds). While the expert-monitoring application relies on the analysis of data collected over a long period (i.e., day, week, and month), thus not requiring a real-time analysis, the self-monitoring application aims to let the user be aware on their current condition/risk to get the proper information in real-time in order to act if alerted.

The self-monitoring application to achieve the objectives described is based on the characteristics of the users (i.e., age, gender, type of photo, etc.) and on the data collected by the sensors. These data are processed to (i) define the user profile (i.e., specific thresholds and risk factors) and ii) calculate the exposure. Note that the user profile is calculated on the mobile application since the user data are stored in the mobile device while the exposure calculation is implemented on the wearable device using the sensor data and then sent to the mobile application.

## 4. Data Utility

The two applications introduced in section 3 have different users and, in order to provide them with high-quality output at the right time and in the right place, these applications must select proper input data. For this reason, it is possible to associate each application with requirements relating to the data of interest and their granularity, as well as to the quality of the service (e.g., responsiveness to the request and availability). To represent these differences and support different applications in a customized way, we introduce the concept of *data utility*.

Data utility can be defined as the relevance of data for the usage context (Cappiello et al., 2017). Relevance is evaluated by considering the capability of the source to satisfy non-functional requirements (i.e., data quality and QoS properties) of the task using the data. Since data utility depends on the application/user that aims to access data for a specific goal, its assessment can be theoretically performed only when the usage context is defined. However, it is possible to identify and assess some dimensions in order to provide an objective estimation of the data source utility level, the so called *Potential Data Utility (PDU)*. The potential data utility provides an estimation of the quality level of the data contained in the whole data source. Note that the potential data utility and the data utility coincide when the user/application aims to use the entire data source as it is offered. As soon as the usage context is related to only a portion of the data source, the data utility must be assessed. However, the potential data utility can be seen as an aggregated reliability index of the data source. In order to assess the data utility, a set of relevant dimensions must be defined. In the following sections, data quality and QoS models are presented.

### 4.1. Data Quality Model

Data quality is often defined as “fitness for (intended) use” (Batini and Scannapieco, 2016), that is, the capability of a dataset to be suitable for the processes/applications in which it has to be used. Data quality is a multidimensional concept since different aspects of the analyzed data must be considered. Such aspects are modeled through data quality dimensions that are defined to analyze specific issues and that are assessed through determined metrics. The literature presents many data quality dimensions but, traditionally, the most used ones are

- *Accuracy*: the degree to which a value *v* is close to a correct value *v*′ (Redman, 1996)
- *Completeness*: the degree to which all the values are present in the considered dataset
- *Consistency*: the degree of adherence to logical rules that link two or more attributes of the considered dataset
- *Timeliness*: the extent to which the age of data is suitable for the task at hand (Wang and Strong, 1996).

Note that the data quality model (i.e., the list of considered dimensions and the metrics for evaluating them) depends on the type of data source. For example, if we consider sensor networks, and therefore a scenario like the one considered in this paper in which the sources generate data streams, it is necessary to consider that the dataset *DS* is an infinite sequence of elements *DS* = (*X*_1_, *t*_1_)(*X*_2_, *t*_2_)…, (*X*_*m*_, *t*_*m*_) in which *X*_*m*_ is, for example, the set of values detected by the sensors on a wearable device at the moment *t*_*m*_ (Klein and Lehner, 2009). The model defined for data quality management relies on the concept of “data quality windows” for which data quality metadata are evaluated by dividing the stream in windows and assessing the quality of the *k* values included in a window. In this context, the metrics related to completeness, consistency, and timeliness do not change, while for the assessment of accuracy the maximum absolute systematic error *a* must be defined and a value *v* is correct if the expected value *v*′ is in the range [*v* − *a*; *v* + *a*] (Klein and Lehner, 2009). Accuracy is important, but it has to be considered together with *Precision* is the degree to which repeated measurements show the same or similar results. Precision is usually estimated by considering the standard deviation and might be an additional information to understand the stability of the measurement process. In fact, situations in which data are not accurate but precise may not always reveal malfunctioning sensors but also a plausible slow change in the observed phenomenon (for example, the expected temperature is increasing).

### 4.2. QoS Model

In the present work, the QoS model includes the following dimensions, which are the most commonly used in evaluating Quality of Service:

- *Availability*: it can be defined as “The ability of a functional unit to be in a state to perform a required function under given conditions at a given instant of time or over a given time interval, assuming that the required external resources are provided” (ISO/IEC, 2010). It usually shows the percentage of the time that the service is up and operational.
- *Response time*: it is the amount of time (usually expressed in seconds or milliseconds) the platform takes to provide the output of a specific request.
- *Throughput*: it is generally defined as the total amount of work completed in a given time. Considering data transmission, it refers to the data transfer rate.
- *Latency*: it is the time interval taken to transmit data between two points in a network.
- *Volume*: it is defined as the amount of disk space or the number of entries in a database.

The evaluation of all these dimensions requires a monitoring platform to provide this information as metadata associated with the data source.

### 4.3. Data Utility in Use

In our approach, each dataset *DS* is firstly associated with a *Potential Data Utility* vector:

(1) $$\begin{array}{l} PDU=\left(qd_{1},qd_{2}...qd_{N}\right) \end{array}$$

in which each value *qd*_*i*_ provides an estimation of a data quality or QoS dimension. As stated above, PDU is a set of metadata that profiles the source without considering the usage context. In this way, PDU provides aggregated information that helps users to understand the reliability of the dataset. PDU can thus be a first driver in the selection of sources if similar datasets are available.

As mentioned above, as soon as the context of use is related to a portion of the data source, it is necessary to evaluate the data utility. In fact, when a user aims to search for a dataset, they will define their functional and non-functional requirements. The former define the part of the available dataset that the user intends to access. Considering our scenario, in the self-monitoring application, the user could be interested only in the values collected in the last 10 min, while, for the expert monitoring application, the user can specify an interest for the data referred to a specific class of customers (for example, characterized by a specific profile, such as age or gender). Moving to the non-functional requirements, they refer to the constraints relating to a series of data quality/QoS dimensions (e.g., response time less than 5 s) considered relevant for the application/process in which the data are used. For example, accuracy, precision, completeness, and consistency are relevant dimensions for both applications, while timeliness is likely to be relevant only in the self-monitoring application where up-to-date data are needed. The description of the application together with the specified requirements define the usage context. In this second phase, the source can be associated with the *Data Utility* vector (DU) for a given usage context. DU informs users about the suitability of the dataset in satisfying their requirements. Note that PDU and DU overlap if the application/user asks to access the whole dataset, while DU has to be reassessed if the usage context considers a dataset *DS*′ ⊂ *DS*.

At the run time, data utility should be periodically assessed in order to detect changes in the quality of data or service. In our approach, if the utility decreases below a certain threshold, one or more adaptation actions are triggered as described in section 5 with the goal of maintaining the data utility at a satisfactory level.

Note that, especially for what concerns the QoS criteria, data utility is dependent on the locations in which data are stored and consumed. In Fog computing, response time, for example, can significantly vary considering datasets in the cloud and datasets in the edge. By taking advantage of the ability to manage datasets in the edge and in the cloud and to move data between the different layers of the Fog computing environment, it is possible to trigger an adaptation action to continuously meet the requirements expressed. In section 5, such adaptation actions are formally defined and discussed.

## 5. Adaptation Actions for Data Management

A key feature of the proposed approach, in addition to the possibility to express the user requirements in terms of data utility, is to enforce the proper satisfaction of such requirements by enabling a set of adaptation actions that can be enacted to solve or prevent violations of the requirements. In particular, the actions considered in our approach refer to actions for moving or copying data, actions for improving the quality of data, and actions for transforming data to support or speed up data analysis.

**Figure 4:**
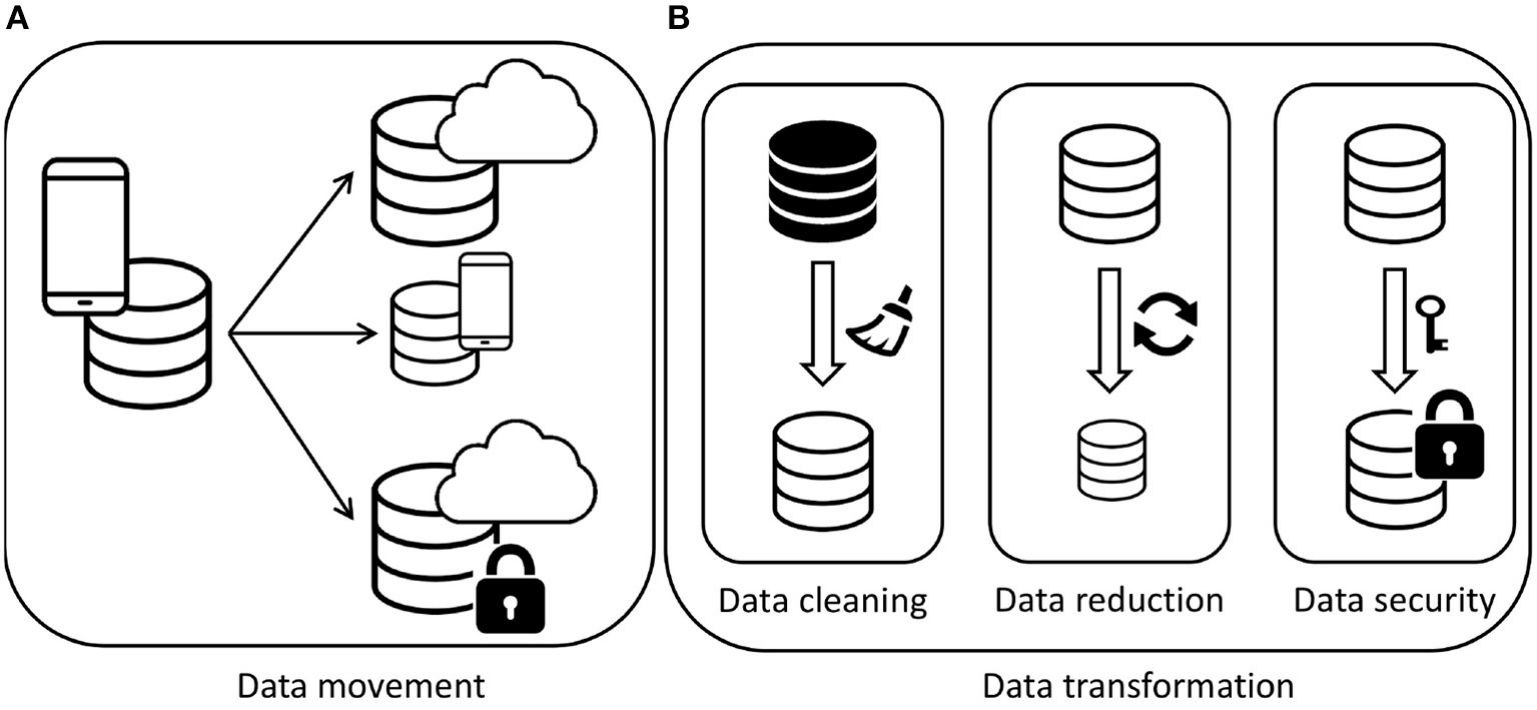
Adaptation actions. **(A)** Data movement. **(B)** Data transformation.

Generally speaking, we refer to the actions that can be enacted to manipulate the data sources in a fog scenario as *adaptation actions*, which are composed of a set of atomic *tasks* T=DMT∪DTT, where (i) DMT are data movement tasks, and (ii) DTT are data transformation tasks (see Figure 4).

In sections 5.1 and 5.2, we illustrate the atomic tasks for adaptations actions^4^.

### 5.1. Data Movement Tasks

Data movement implies the transfer or the duplication of (a portion of) a dataset from a storage resource to another one. For instance, data can be moved from the edge—where they are generated—to cloud storage, like in the reference example where the data collected on a smartphone are periodically uploaded to the cloud. Generally speaking, a data movement task can be defined as

- The location of the resources involved in the movement from source *a* to destination *b*, which can be classified according to their layer (either in the edge *E* or in the cloud *C*)^5^.
- The kind of movement applied, e.g., movement of data from one resource to another (deleting the previous version) *M*_*ab*_ or creation of a replica on different storage resources *D*_*ab*_.

Considering the possible combinations of resource location and kind of movement, the following eight main relevant tasks are considered in DMT:

- Move/Duplicate from cloud to edge (*M*_*CE*_/*D*_*CE*_): data are moved or copied from a cloud to an edge resource.
- Move/Duplicate from edge to cloud (*M*_*EC*_/*D*_*EC*_): data are moved or copied from an edge to a cloud resource.
- Move/Duplicate from cloud to cloud (*M*_*CC*_/*D*_*CC*_): data are moved or copied from a cloud to another cloud resource.
- Move/Duplicate from edge to edge (*M*_*EE*_/*D*_*EE*_): data are moved or copied from an edge to another edge resource.

Considering the running example, data movement between two edge devices occurs when data collected by one user is moved to another user's device, for example, to share information between family members. In addition, data movement between edge and cloud occurs when data about a user's activity is moved from their device to cloud storage. There, the data can be aggregated with the data of other users to be analyzed in the future by an expert.

The Data Movement tasks introduced in this section are categories of tasks. It means that they represent a generic movement according to the type of resources involved. In fact, categories are useful in aggregating together actions that are likely to have similar impacts when applied in a specific context. As discussed in Plebani et al. (2018), when implemented in a specific scenario and according to the actual resources available, one or more instances for each category might be instantiated to represent all possible movements between all the possible resources. Considering the example of Figure 3, two instances for the class *M*_*CE*_ and *M*_*EC*_ are created (since we have two edge devices connected with a cloud resource); similarly, two instances for *M*_*EE*_ are generated, and no tasks of type *M*_*CC*_ are available since only one cloud location is available in the scenario. The set of instantiated tasks depends also on the policies defined in the application context. For instance, in case we want to enable only movement in one direction, from edge to cloud, the *M*_*CE*_ tasks are not instantiated.

### 5.2. Data Transformation Tasks

While data movement tasks affect the location of a dataset, in the case of data transformation tasks a single data source is affected. In particular, the goal of this type of tasks is to produce a modified version of the dataset applying some filtering and/or transformations. More precisely, given a dataset *DS* where the degree (the number of domains) is *deg*(*DS*), and the cardinality (the number of tuples) is *card*(*DS*), a transformation task dtt∈DTT of a dataset *DS* produces a new dataset *DS*′

(2) $$\begin{array}{l} dtt\left(DS\right)\to DS^{\prime } \end{array}$$

On this basis, the transformation tasks could affect both the intensional and the extensional schema. In fact, in this class of tasks, data aggregation as well as data projections are included. In the first case, a set of tuples can be reduced to one (for example, by averaging a series of observations), which thus reduces cardinality. In the second case, some columns are removed as they are considered irrelevant or—in case of privacy problems—not accessible, thus reducing the degree of the dataset. Here we define three types of transformation tasks, which have different effects on the degree and the cardinality. In particular, we are interested in this work in three main sets of transformations: (i) *data-cleaning-related transformations*, (ii) *performance-related transformations*, and (iii) *security-related transformations*,

**Data Cleaning related transformations** aim to improve the quality of the data. In a data stream the cleaning tasks can be:

- **Inputting missing values**: missing values can be fixed by considering different techniques, such as (a) using unbiased estimators that estimate missing values without changing characteristics of an existing dataset (e.g., mean and variance), (b) using mean or median to replace missing values, or (c) adopting a specific distribution.
- **Outlier management**: an outlier can be generated since (a) the value has been incorrectly observed, recorded, or entered in a dataset, or (b) the value is correct but represents a rare event. The cleaning task is responsible for discovering outliers and for deciding between rare data and data glitches. Data glitches should be removed while rare events should be highlighted.

The former task mainly improves the completeness without negatively affecting the accuracy. In fact, the inputting techniques try to insert acceptable values. The latter task has instead a positive effect on accuracy and precision when data glitches are discovered. Note that a data cleaning task affects only the extensional schema of the data source, while the intensional schema is preserved. In fact, the improvement of data quality operates at record level [*deg*(*DS*′) = *deg*(*DS*) and *card*(*DS*′) = *card*(*DS*)]. In summary, it is possible to enact a specific cleaning task on the basis of the dimension that caused a violation. The enactment of this task is time consuming and expensive in terms of computational power. Its execution performance might therefore be different in the edge or in the cloud.

In the considered scenario, data cleaning is a transformation technique that could be enabled both in the self- and expert monitoring when data quality requirements are not satisfied.

**Performance-related transformations** can be enacted to improve the performance of the enactment of an adaptation action. One of the main issues with adaptation actions is the management of high volumes of data that can generate delays and performance issues. For instance, the volume of data collected by the IoT and sensors at the edge makes the data movement for analysis from the edge to the cloud difficult and time consuming, and, in addition, it might introduce critical delays. For this reason, it is important to reduce the size of the data to be moved in order to make this task more agile. Performance related transformations are:

- **Aggregation**: the content of a data storage is reduced using aggregation operations (e.g., average, maximum, and minimum) summarizing several tuples.
- **Reduction**: the data volume is reduced by exploiting relations among data.

Both performance transformations reduce the volume of the data that must be transferred from the source to the destination.

**Aggregation** applies classical operators (e.g., average, maximum, minimum, and sum) to several events collected in the dataset. The effect is to reduce the volume of the dataset while affecting the level of detail contained in it. Aggregation is not reversible (it is not possible to obtain the original data from the aggregated set). This transformation aims to reduce the cardinality of the dataset but it does not affect the degree (*deg*(*DS*′) = *deg*(*DS*) and *card*(*DS*′) < *card*(*DS*)).

**Reduction** is based on the assumption that the information stored in a dataset may contain related items. In literature, data reduction is performed mainly on a single signal by varying the sampling frequency based on the variability of the monitored variable (Trihinas et al., 2018). We additionally propose to exploit relations between different variables, which can be expressed as dependencies among the values of related attributes. For example, relations are those expressed through functional dependencies between the data values in a dataset. Functional dependencies are also used to check consistency in the dataset. For instance, it is possible to obtain the city and the country where a user resides from the postal code. A causal relation between attributes in a dataset can be expressed through an association rule *A*⇒*B* expressing that the value of attribute B depends on the value of attribute A. Association rules are effective to represent relations between non-numerical attributes that can get a limited number of values. For numeric values, we instead apply regression functions to represent the dependencies between a dependent variable and a set of correlated variables from which it is possible to calculate its value. Relations can be both explicit and implicit. Explicit relations are declared by the data owner, who also provides the association rules for non-numerical attributes or the regression model for numerical values. Instead, implicit relations can be detected using data mining and machine learning techniques. While several approaches exist for extracting association rules between attributes of a dataset, the detection of dependencies between numerical values is not trivial (Peng and Pernici, 2016). Reduction enables to regenerate the original information, although with some approximations. It might reduce both the degree and the cardinality (*deg*(*DS*′) ≤ *deg*(*DS*) and *card*(*DS*′) ≤ *card*(*DS*)).

**Security-related transformations** aim to satisfy security constraints that might affect a data source when moved from one location to another. As an example, when data are collected inside the user device, they contain the information that is needed to identify a specific person. When these data need to be moved and stored outside the device, privacy constraints might require that sensitive information must be hidden so that unauthorized customers cannot access it. Security-related transformations include the following.

- **Pseudonymization**: data are manipulated to substitute identifying fields within a data record with artificial identifiers.
- **Anonymization**: data are manipulated to remove all possible identifiers.
- **Encryption**: the data contained in a data storage are manipulated using encryption algorithms to make them unreadable to unauthorized users.

None of the security-related transformations affect the cardinality of the dataset (*card*(*DS*′) = *card*(*DS*)). Instead, while pseudonymization and encryption do not affect the degree, anonymization might remove some of the attributes from the dataset (*deg*(*DS*′) ≤ *deg*(*DS*)).

### 5.3. Defining Relevant Adaptation Actions

Based on the knowledge of the available datasets, their location, their relations, and the privacy and security constraints, there are two main aspects that the system design has to take into account to define suitable adaptation actions based on the atomic tasks illustrated above:

- Defining the adaptation actions that are relevant in the considered application domain
- Identifying the most suitable action to perform in a given time.

In this section, we focus on the first problem, discussing the relevant aspects that must be taken into consideration when adaptation actions are to be finalized. About the second issue, section 6 discusses how the goal-based approach is adopted to drive the execution of the adaptation actions with the aim to improve data utility.

The different kinds of tasks introduced in this section are the building blocks for composing an adaptation action. In fact, an adaptation action can include one or more tasks. More formally, an adaptation action aa∈AA in a specific application context is defined as a tuple:

(3) $$\begin{array}{l} aa=<t_{a},ManT,OptT> \end{array}$$

where

- *t*_*a*_ ∈ *T* is the main task of the action and can be either a data movement or a transformation task.
- ManT∈DTT is a set of mandatory tasks that are always executed with the main task.
- OptT∈DTT is a set of optional tasks that can be associated with the main task.

Both mandatory and optional tasks are transformations applied to the dataset for complying with the security requirements or for improving the effect of the main task. As an example, different privacy and security constraints might apply to each location, and a data movement action could therefore also require some security-related data transformation. For instance, due to the privacy regulations, data stored on the cloud must be anonymized, and data collected on a smartphone should thus be made anonymous by removing any direct reference to the user (e.g., userid and name) before moving them from the user's device to the cloud. According to this, we can define an adaptation action *aa*_1_ = {*M*_*EC*_, {*anonymization*}, {*reduction, aggregation*}} composed of a main task *t*_*a*_ = *M*_*EC*_, which moves the data collected by a wearable device from the smartphone of the user to the cloud storage. The mandatory task *anonymization* forces to anonymize the data when the movement is performed. Finally, *Opt* = {*reduction, aggregation*} defines as optional the tasks reduction and aggregation, both reducing the volume of data to be moved from the device to the cloud, and this consequently improves latency and reduces cost.

As already defined, adaptation actions might affect both the content (data transformation tasks) or the location (data movement tasks) of a dataset. The argument of an adaptation action can be a whole dataset or a subset of it. As an example, when the cloud is fed with the data from the user's device, the action could include either a *M*_*EC*_ or a *D*_*EC*_. Considering our scenario, when the storage on the device is almost full, moving data from the edge to the cloud might require emptying all the collected data stored in the edge to be saved in a cloud resource. However, the requirements of the running applications might be in conflict with this strategy since some data might be useful locally. For instance, some of the data should be kept locally to support the self-monitoring application. Observing the past executions of the application it is possible to provide information on the typical behavior expected by the system. Here, we focus on two main aspects:

- Relevant data: not all the data collected by the sensors at the edge are used locally. When deciding which data to move from the edge to the cloud and vice versa, we should take into account the frequently accessed data. This information is relevant to improve the performance of the data retrieval. As an example, when the storage resource at the edge side is full, we should move some data to the cloud. In doing so, we can select the data that are less likely to be used in the near future and keep the other data on the device to keep the data retrieval latency low.
- Device behavior: in a fog environment, we are often subject to unreliable connections between the cloud and the edge. Consequently, the user device can be offline at some point, making the communication between the cloud and the edge impossible. Observing the typical behavior of the devices in terms of connectivity with the cloud, we can prevent connectivity issues by using this information when deciding where to place the data. As an example, statistics and aggregation of monitoring data are usually performed and stored in the cloud. A customer who wants to access statistics needs to have an active connection all the time. If a permanent active connection is not ensured, our approach can improve the performance by saving an extract of the statistics back to the edge, thus making it accessible every time to the customers, even when connectivity is not present.

## 6. Improving e-Health Monitoring With Data-Utility Driven Adaptation Actions

The main goal of each data provider is to offer its services by meeting consumer demands in terms of data usage. However, Fog computing is a dynamic environment in which the performance of the fog nodes can deteriorate and the connections between the nodes are not reliable or simply not durable due to the mobility of the fog nodes. This section describes the part of the approach, proposed in this document, which allows the provider to meet the users' data requirements in such a dynamic environment, choosing the best adaptation action.

We define a goal-based modeling language in order to specify (i) user requirements, (ii) the adaptation actions that can be implemented, and (iii) the link between adaptation actions and user requirements. The information modeled with this language is the basis for selecting the best adaptation action.

In the literature, several goal-based modeling languages are defined (Horkoff et al., 2015). However, as far as we know, no goal-based modeling language allows the definition of adaptation actions and their impact on goal models. We based the definition of the language on our extension (Plebani et al., 2018) of BIM modeling language (Horkoff et al., 2012).

### 6.1. A Modeling Language to Link User's Requirements With Adaptation Actions

Non-functional requirements of applications using the same dataset are expressed through the concept of data utility introduced in section 4. For each application, a different set of dimensions is selected, and the desired value is indicated for each dimension. When these requirements are not met, our approach identifies the violations and detects which adaptation actions can be enacted.

We have chosen a goal-based modeling language to represent the requirements since this type of language allows for easy classification of requirements based on users' objectives (goals) across different levels of abstraction. This feature allows the readability of the goal model even by non-technical users.

In a goal model, the *goal* concept represents an objective to be achieved. Formally, the set of goals G in a goal model is defined as

(4) $$\begin{array}{l} G=\left\{<Name,Metrics>\right\} \end{array}$$

where *Name* is the name of the goal and Metrics a set of metrics used to assess the goal defined as

(5) $$\begin{array}{l} Metric=\left\{<Type,Comparator,Measure>\right\} \end{array}$$

where *Type* ∈ *Types* indicates the type of the metric referring to the set of data utility dimensions defined in section 4; *Measure* ∈ IR represents the reference value; *Comparator* ∈ {<,≤,>,≥,=} represents the relation between the observed value and the reference value.

In a goal model, each goal can be decomposed into sub-goals forming a tree structure, where the root element is called root goal. Sub-goals represent a set of objectives that, once achieved, allow the achievement of their parent goal. Root goals specify the main objectives (requirements) of users and, therefore, must be satisfied.

**Figure 5:**
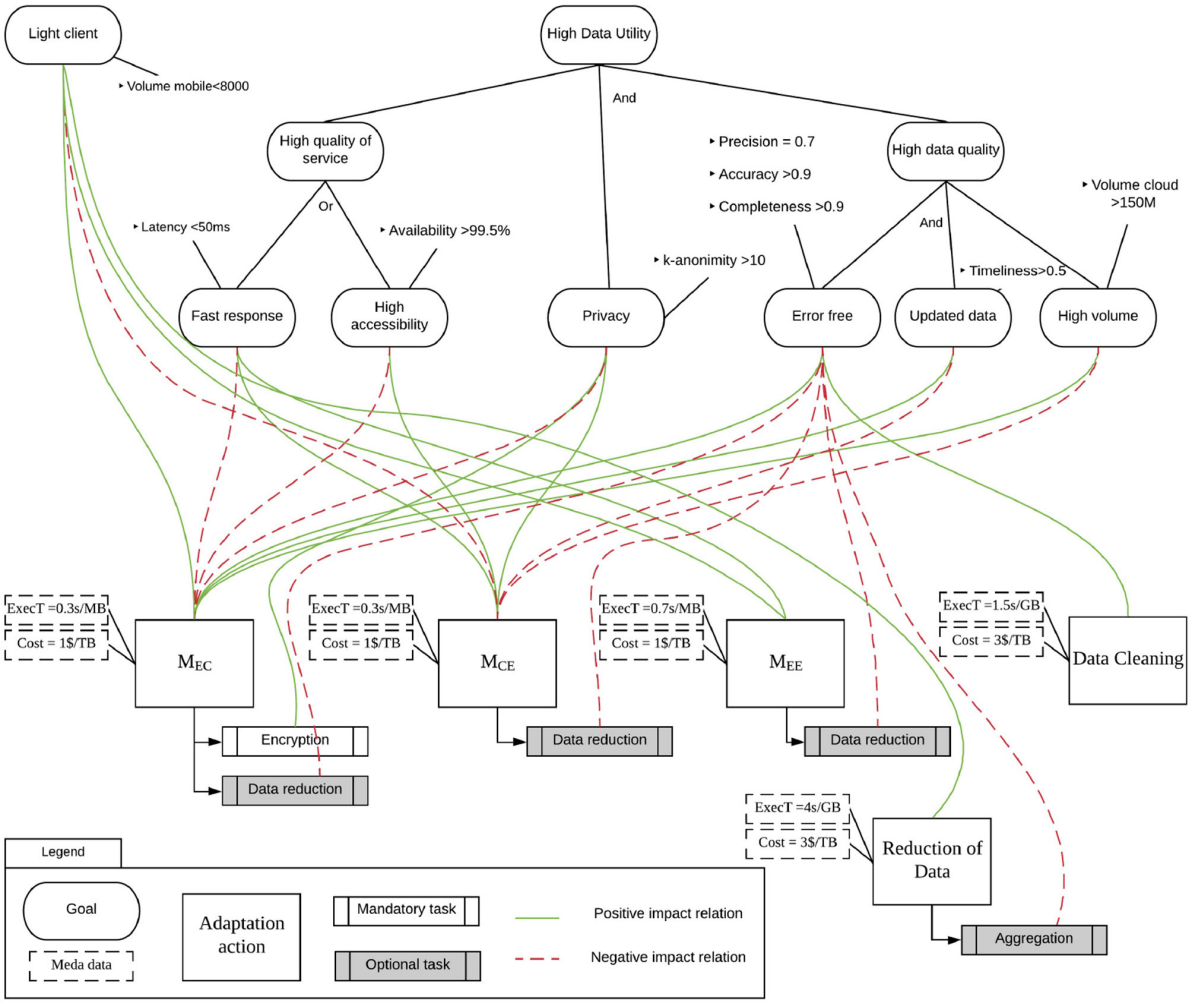
Example of a goal model.

The upper part of Figure 5 provides a graphical representation of the goal-based model formalization applied to the self-monitoring application described in section 3. Each ellipse represents a goal that is linked to measurable metrics that are used to specify when a goal is achieved. For example, the “High availability” goal is linked to the “Availability >99.5%” metric, which means that the goal is considered achieved if the availability of the service is more than 99.5%. The diagram shows two goal trees. On the left, the goal tree is composed only of a “Light client” goal, which specifies that the user wants to limit the volume of the data stored locally in the edge device. On the right, the target tree represents the user's data utility requirements, and it is more complex since it contains a decomposition of the goal model. For example, “Quality of Service” is a parent goal, while “Fast response” and “High accessibility” are its sub-goals. Goal models define two types of decomposition:

- AND-decomposition: all sub-goals must be achieved to achieve the parent goal;
- OR-decomposition: at least one sub-goal must be achieved to achieve the parent goal.

It is worth noting that a violation of a single metric—and therefore of the linked goal—may not imply the violation of the root goal. For example, if the latency of the provided service goes above 50 ms while its availability is greater than 99.5%, then the user's requirements are not violated since the two metrics are linked to two goals which OR-decompose the parent goal. The goal model in the figure requires only one of them to be achieved.

The set of all decompositions are represented by the set Decompositions that is defined as

(6) $$\begin{array}{l} Decompositions=\left\{<g,sub,type>\right\} \end{array}$$

where:

- *g* ∈ *Goals*, is the parent goal;
- sub∈P(Goals), belongs to the power set (i.e., the set of all possible combinations of the elements) of goals and contains the children goals that decompose the parent goal;
- *type* ∈ {*and, or*} is the type of decomposition.

For example, the goal “High data utility” is AND-decomposed in three goals: “High quality of service,” “Privacy,” and “High data quality.” This results in the following decomposition:

{*High data utility*, {*High quality of service, Privacy, High data quality*}, *AND*}

The lower part of the model in Figure 5 represents the adaptation actions that can be enacted in this running example. The adaptation actions modeled define movement and duplication between edge devices to cloud storage, and data transformations. Adaptation actions are represented by boxes with a label that defines the source, the destination, and the type of action. All tasks composing the adaptation action are linked to goals with relations that specify the positive or negative impact that their enactment has on the achievement of the goal. For example, the adaptation action *M*_*EC*_, according to the definition given in section 5.3, defines an action in which the main task concerns the movement between the sensors and the cloud. The action is linked with a positive impact on the “High volume” goal since the movement of data in the cloud has a positive impact on the linked metrics. Indeed, storing data in the cloud, instead of in an edge device, enables the storage of a higher volume of data.

As specified in section 5.3, an adaptation action is associated with optional and mandatory tasks. In the modeling language proposed in this paper, these tasks are represented with a box with double borders on both sides associated with an action: mandatory tasks have a white background, while optional tasks have a gray background.

Each action is associated with a link to the goals that represents the impact of the action over the goal satisfaction. Impacts can be positive or negative. For each action, Pos⊆Goals represents the set of goals that receive a positive impact when the main task is executed, and Neg⊆Goals represents a set of goals negatively impacted. Also, Pos∩Neg=∅. Optional and mandatory tasks inherit impacts of the adaptation actions they are linked to. If they provide additional or different impacts, links to the affected goals are represented in the model. For example, the adaptation action *M*_*EC*_ has one mandatory task “Encryption,” which specifies that data can be encrypted before moving them. Such task has a positive impact on the “Privacy” goal since the encryption will prevent the disclosure of personal data. In this example, the adaptation action and the linked task have an opposite impact on the “Privacy” goal. If this is the case, the impact of the task overcomes the impact of the main task of the adaptation action. Impact relations can be designed by experts or learned/refined automatically by observing the effects of executing a task on the metrics linked to goal model.

As specified in section 5.3, adaptation actions are enriched with metadata. These metadata in our model specify two aspects: the execution time of the action and the economic cost of its execution. Metadata are represented in the model as dashed boxes. For example, the adaptation action *M*_*EC*_ has attached two metadata specifying the execution time (i.e., an estimation of 0.3 s per MB) and the cost (i.e., 1$ per TB). According to this, for each action we can define an element of the metadata set metaData∈MetaData as

(7) $$\begin{array}{l} metaData=<metaType,\text{IR}> \end{array}$$

where *metaType* ∈ {*ExecT, Cost*} is the set of possible metadata. Optional and mandatory tasks can change the metadata of the action to which they are linked.

Formally, we define a task t∈T, including impact relations and meta data:

(8) $$\begin{array}{l} t=<taskType,Pos,Neg,MetaData> \end{array}$$

where *taskType* is the type of task, as defined in section 5, Pos and Neg are the positive and negative impacts of the task, and MetaData are the metadata associated with the task.

At this point we can define a goal model GM :

(9) $$\begin{array}{l} GM=<G,Decompositions,AA> \end{array}$$

### 6.2. Creation of A Goal-Based Diagram for Supporting Adaptation Action Selection

The creation of a diagram based on the modeling language defined in section 6.1 consists of the following phases:

- Creation of the goal model structure that represents user requirements;
- Specification of the adaptation actions that can be enacted, complemented with mandatory and optional tasks and metadata;
- Specification of the impact of adaptation actions and tasks on the goal model.

In terms of the specification of the goal model, users specify their requirements based on their objectives. As already described before, we chose a goal-based modeling language since it can be used to represent requirements at different levels of abstraction. Users can thus express abstract requirements in the upper part of the goal model, while they can specify more concrete requirements on the lower part nearer to the leaves and up to the definition of the reference values for the metrics.

The specification of the adaptation actions largely depends on the infrastructure and on the resources. In section 5.3, we defined a set of classes that will be instantiated according to the actual context of execution. Instances of adaptation actions are not specified in Figure 5 due to space constraints. For example, the adaptation actions *M*_*EE*_, which consists in the movement between two edge devices, will be instantiated by generating two actions for each possible pair of edge elements authorized to exchange the data.

### 6.3. Automated Selection of Adaptation Actions

The main objective of the proposed goal-based model is to provide a method for identifying which is the best action to be enacted in case a goal is violated. In fact, when the violation of a metric prevents the achievement of the top goal of a goal tree, the model supports the selection of the best adaptation action to be implemented in order to remove the violation. This is reflected by the connections between the upper and the lower layers of the model. A software component has been developed^6^ supports this identification by exploring the tree and the positive/negative impacts. The selection is divided into the three phases described below. The software requires the implementation of a monitoring system that identifies the violations of user requirements.

**Phase 1: Selection of the relevant adaptation actions** The first step is to identify the set of adaptation actions that can be enacted to solve a violation. In this step, impacts are used to identify the actions with a positive effect on the violated goal. For example, if the latency goes above 50 ms, the requirement specified by Figure 5 for the sub-goal “Fast Response” is violated. Two adaptation actions are then selected: *M*_*EE*_ and *M*_*CE*_.

This phase executes two algorithms: the first one identifies the violated goals (Algorithm 1), while the second one identifies the adaptation actions that have a positive impact on them (Algorithm 2).

**Algorithm 1:** Identify unsatisfied goals

1: ∀(g,Metrics)∈G
2: $\forall \left(t_{m},C_{m},M_{m}\right)\in Metrics$
3: if $\exists \;m_{e}=\left(t_{me},m_{me}\right)\in Me$ s.t. *t*_*me*_ = *t*_*m*_ ∧ ¬*apply*(*m*_*me*_, *c*_*m*_, *m*_*m*_)
4: then g is violated

**Algorithm 2:** Identify adaptation actions

1: $\forall \left(t_{a},ManT,OptT\right)\in AA$
2: if ∃g∈VG s.t.*g* ∈ (*pos*(*t*_*a*_)∪*poss*(*ManT*)) \ *negs*(*ManT*)
3: then $\left(t_{a},ManT,\emptyset \right)\to SelectedAA$
4: ∀*o*_*p*_ ∈ *OptT*
5: if ∃g∈VG s.t.*g* ∈ *pos*(*o*_*p*_)
6: then $\left(t_{a},ManT,\left\{o_{p}\right\}\right)\to SelectedAA$
7: $\forall selectedAA=\left(t_{a},ManT,OptT\right)\in SelectedAA$
8: if ∃g∈VG s.t. *g* ∈
9: (*neg*(*t*_*a*_) \ (*poss*(*ManT*)∪*poss*(*OptT*)))∪
10: (*negs*(*ManT*) \ *poss*(*OptT*))∪
11: *negs*(*OptT*)
12: then remove *selectedAA* from SelectedAA

Algorithm 1 requires as input the goal model GM={G,Decompositions,AA} defined in Equation (9), and a set of measures $Me=\left\{T_{me},M_{me}\right\}$, where *T*_*me*_ ∈ *Types* and *M*_*me*_ ∈ IR. Measures are generated by a monitoring system that continuously checks the system targeted by the goal model.

where *apply*(*m*_1_, *c, m*_2_):*boolean* is a function that applies the operator c∈Comparators to the two measures *m*_*me*_, *m*_*m*_ ∈ IR.

Algorithm 1 inspects all goals in the goal model (Line 1). For each metric in each goal (Line 2), the algorithm checks if it exists a measure that has the same type and violates one of its metrics (line 3). If this is the case, the goal is considered to be violated (Line 4).

Given a goal model GM={G,Decompositions,AA} and the set of violated goals VG identified thanks to Algorithm 1, Algorithm 2 identifies the relevant adaptation actions in AA.

In Algorithm 2:

- *pos*(*T*):*Goals*, where T∈T is a function that returns the set of goals positively impacted by the task in input;
- *neg*(*T*):*Goals*, where T∈T is a function that returns the set of goals negatively impacted by the task in input;
- $poss\left(T^{\prime }\right):Goals$, where $T^{\prime }\subseteq T$ returns the union of all goals positively impacted by all tasks in the set received as input;
- $negs\left(T^{\prime }\right):Goals$, where $T^{\prime }\subseteq T$ returns the union of all goals negatively impacted by all tasks in the set received as input.

Algorithm 2 considers each adaptation action (Line 1) and verifies if there exists a violated goal that belongs to the set of goals that are impacted positively by the main task or the mandatory tasks of the adaptation action (Line 2). If this is the case, the adaptation action is added to the set of selected actions *selectedAA*. The selected action set includes only the main task and the mandatory tasks (Line 3). Please notice that, in Line 2, we subtract the goal negatively impacted by *ManT* tasks since impacts of mandatory and optional tasks override impacts of the main task (see section 6.1).

Additionally, for each optional task of the action (Line 4) the algorithm verifies if they have a positive impact on a violated goal in VG (Line 5). If this is the case, the adaptation action is selected, including the optional task.

Finally, for each action in *selectedAA* (Line 7), the algorithm checks if the action has a negative impact on one of the violated goals (Line 8–11), removing it from the set (12). Similarly to Line 2, also in this case the algorithm subtracts from the set of goals negatively impacted by the main task the positive goals of mandatory and optional task (Line 9). Line 10 specifies that an impact relation of an optional goal overrides an impact relation of a mandatory goal. We chose this criterion since we assume optional tasks are chosen to improve the behavior of the adaptation action (and its mandatory tasks).

**Phase 2: Prioritization of the adaptation actions** The second phase consists of the prioritization of the adaptation actions selected in the first phase based on their metadata and on the strategy selected by the user. Strategies are optimization functions that consist of the (set of) metadata that the user would like to minimize or maximize. For example, they can define as a strategy the minimization of the costs or the minimization of the execution time. Once the strategy has been defined, the selected adaptation actions will be ranked and the enactment of the action with the highest score suggested.

The selection, and consequently the enactment, of an adaptation action brings to the system a new configuration where data have been moved, copied, and/or transformed to resolve a violation. Algorithm 2 selects the actions that have a positive impact on violated goals. It is worth noting that the correctness of the output, i.e., whether the selected adaptation action positively impacts the violated goals as expected, is based on the correctness of the input, i.e., the goal model analyzed.

**Phase 3: Update of impact relations** After the enactment of an adaptation action, the framework will periodically check the metrics and update the impact relations based on the performance of the system after the enactment.

     □

Adaptation actions are selected and enacted one at time, with a time span between two enactments that is sufficient to measure the impact of the action on the goal model. Every time an action is selected by Algorithm 2, it is enacted, and metrics of the goal model are measured in order to detect the impacts of the adaptation action and, possibly, update its impact relations.

Algorithm 2 selects only adaptation actions with a positive impact on the goals violated in the model. This ensures that, granted the correctness of the goal model, the system is led to a configuration that resolves (or reduces) the violations. This is guaranteed by lines 1–3 of the Algorithm 2 where only actions with positive impacts on violated goals are selected. Lines 4–6 enrich this set of actions with actions with optional tasks, having at least a positive impact on violated goals, while lines 8–11 remove from the set adaptation actions with negative impacts on the violated goals. This last step aims to avoid side effects in the enactment of an action.

It is worth noticing that the method described in this section is successful only if the goal model is generated in a proper way. First of all, the goal model must contain all the relevant requirements of the application/user. It is very important to be able to capture whether the current configuration does or does not satisfy the users' needs. Second, the treatment layer of the goal model must contain all and only the actions applicable in the context. This is very important for avoiding the system to be driven in undesired configurations. This depends on a proper definition of the rules for where and how it is possible to move data from a location to another set by the data administrator. Finally, impacts linking the treatments to goals must properly represent the effects of enacting the selected action. For this, the expert's knowledge is very relevant, but might not be enough. To help in the definition and refinement of the impacts, real effects are analyzed to improve the model by updating impacts according to what observed at run-time. Correct impacts enable us to predict the positive and negative effects of an action and to avoid disruptive decisions. A sound model thus provides all the elements to detect and react to violations taking informed decisions.

The described framework considers only the goal model for a specific user at a time. In future work, we will consider multi-users scenarios, where multiple goal models, potentially defining conflicting requirements, will be evaluated. In this case, two solutions can be adopted: (i) a centralized decision system that will have the control on all the goal models and it will select the best adaptation action and (ii) a distributed decision system that will divide the responsibility for the selection of the best adaptation actions among all its participants.

### 6.4. Using the Goal-Model in the Healthcare Scenario

Referring to our motivating example discussed in section 3 and to the goal model shown in Figure 5, we explain the usage of the proposed approach through some examples.

Let us suppose that the telecommunication provider of user A's smartphone is experiencing traffic congestion. This negatively impacts both latency and availability, which decrease to the point that they violate both the *Fast response* and the *High accessibility* goals. Consequently, the goal model can be used to find compensating actions capable of fulfilling the requirements once again. Based on the goal model, the adaptation action *M*_*CE*_ (i.e., move data from cloud to edge) is selected, as it has a positive impact on both violated goals, and the negative impact on other goals is negligible. To decrease the time required to perform data movement, the *Data reduction* transformation is additionally applied while moving data from the cloud to user B smartphone. Although *Data reduction* has a negative impact on the *Error Free* goal, the effect of such impact is not sufficient to violate that goal. Its execution thus has a positive effect.

Let us now consider that the volume of UV light measurements on user A's smartphone exceeds 10,000 samples, violating the *Light client* goal. Based on the goal model, the adaptation actions labeled *M*_*EC*_ and *M*_*EE*_ are selected, as they both have a positive impact on the *Light client* goal. In terms of the smartphone of user B in proximity with the one of user A, the adaptation action *M*_*EE*_, which moves data between the two devices, is enacted, as it has no negative effects on the other goals, and its cost is lower or equal to the one of every action of the *M*_*EC*_ class. .

Let us also consider that it is possible to experience issues related to the reliability of values received from the UV sensor. Such problems can be caused by two main reasons: communication problems between sensors and the smartphone and degradation of the sensor's performance due to, for example, low battery or failures. In both cases, the reliability of the UV sensor values decreases to the point that it no longer fulfills the completeness and/or accuracy requirements specified for the *Error free* goal. Consequently, the adaptation actions labeled *M*_*EC*_ and *DataCleaning* are identified as candidates. Since *M*_*EC*_ has a negative impact on the *Fast response, High accessibility*, and *Privacy* goals, it is set aside in favor of *DataCleaning*, which will have only impact on *Fast Response*. In fact, enabling the data cleaning transformation will, on the one hand, take longer to process and display data but, on the other hand, it will try to provide reliable results. Within the “inputting values” features, null values will be detected and (if possible) substituted with reliable values. The “outlier detection” will analyze outliers, that will be removed or substituted with acceptable values if related to data glitches. In any case, it is necessary to underline that, if the quantity of values received is too low, no cleaning operation is possible, and the application should warn users of the system failure.

## 7. Tool Evaluation

The main feature of the software component we developed consists of deciding which is the best adaptation action to be enacted and, consequently, foreseeing the effects of such actions. We therefore executed tests to measure the ability of the software component to perform a choice that leads the system to a configuration that does not violate any goals defined in the goal model.

We simulated typical configurations for the case study and triggered several violations multiple times. We measured how many times the system is brought to a configuration where violations are removed and how many actions are enacted to reach such configuration.

We repeated the test with (i) a growing number of violations, (ii) reduced the number of edge/cloud resources where it is possible to move data, and (iii) reducing the quality of the network connection between resources. We have implemented software optimizations that allow the analysis of available resources and the selection of the best one; however, these optimizations cannot be applied on a network where, especially in a fog environment, the connection may not be stable. We therefore ran an additional set of tests to verify the behavior of the software component when optimizations cannot be applied.

**Figure 6:**
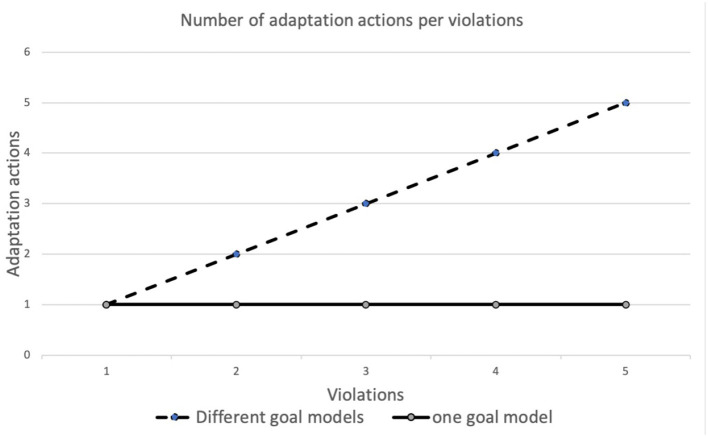
Number of adaptation actions per violation.

Figure 6 shows the number of adaptation actions (Y axis) enacted based on the number of violations detected (X axis) simultaneously. The dashed line shows the situations in which multiple goal models (one for each user/application) are managed. In the test, increasing the number of violations corresponds to the introduction of an additional goal model; at any step, therefore, only one violation per goal model is detected. For example, five violations mean that five goal models (one for each user/application) detected one violation each. In this case, the number of adaptation actions, required to reach a system without violations, is equal to the number of violations received. The software examines one violation at a time and enacts the corresponding adaptation action to solve it. The solid line, instead, shows the behavior with multiple violations on a single goal model. As can be seen, in the experiments one adaptive action is sufficient to resolve all violations. By comparing the two behaviors, we can observe that, for a single goal model, an action can resolve multiple violations. Actions, however, have an impact on the system only at the local level. When multiple goal models are considered, an action must be taken for each goal model that has detected a violation.

**Figure 7:**
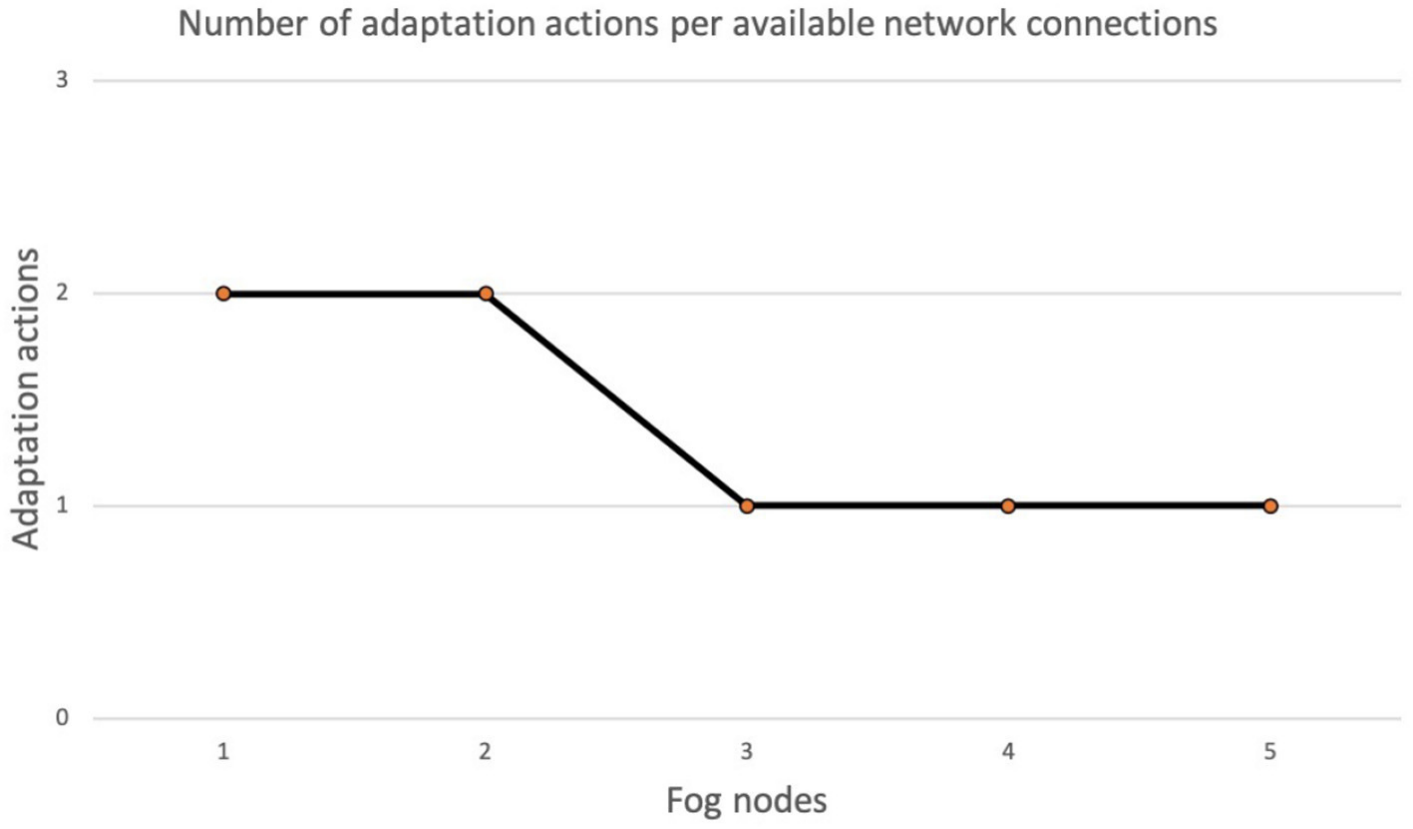
Number of adaptation actions per available network connection.

Figure 7 shows the number of adaptation actions necessary in the event of deterioration of the quality of the network. We simulated the network using virtual connections, each of them with a set of properties, such as latency. The chart in Figure 7 shows in the X axis the number of virtual network connections that can be used to restore a configuration of the system without violations, while the Y axis represents the number of adaptation actions enacted to bring the system to a configuration where no violations are detected. For a large number of available network connections (3, 4, 5) the correct decision is made immediately. For fewer available network connections, the correct decision is made after the second adaptation action. We repeated the experiment several times, but the number of adaptation actions was always the same since the software component uses a deterministic algorithm for the decision.

Summarizing what is shown in this section, the results of the experiments show that the number of violations affects the selection of the best adaptation action. When we deal with a single goal model (solid line in Figure 6), a single adaptation action can solve all violations. Instead, with multiple goal models (dashed line in Figure 6) each violation is triggered by a different node, therefore, the number of adaptation actions needed is equal to the number of violations detected. Concerning the relation between the number of adaptation actions and the quality of the network, the results in Figure 7 have shown that the lower the network quality, the higher the number of adaptation actions that may be needed to restore a configuration of the system with no violations. Network connections in fog environments are unstable and their characteristics change frequently. To know the state of the network, a continuous benchmark would be necessary; however, the impact of this activity would create an excessive overload. The software component therefore tries to implement an adaptation action and waits for the next violation.

## 8. Related Work

The evolution of data management systems in the last year has confirmed that the “one size fits all” approach is no longer valid (Stonebraker and Cetintemel, 2005) and this is also confirmed in the healthcare domain. In fact, nowadays, data intensive applications (Kleppmann, 2017) are not based on a unique database technology (e.g., relational databases) (Prasad and Sha, 2013). Also, the computation is now polyglot (Kaur and Rani, 2015), i.e., different modules are developed with different languages. This trend has been boosted also by the availability of platforms that usually support the micro service architectural style.

Although these new approaches provide a support for an easy development and execution of scalable and reliable solutions, the negative aspect concerns the need for inter-process communications in place of the shared memory access that is heavily affected by the network performances (Dragoni et al., 2017). For this reason, proper data management is required, and the information logistics principles are useful in this context (Sandkuhl, 2008; Haftor et al., 2011). In particular, (Michelberger et al., 2013) identifies different perspectives around which Information Logistics can be studied: e.g., from an organizational standpoint in terms of how to exploit the data collected and managed inside an organization for strategy purposes or how to properly distribute the data in a supply chain management. The so-called user-oriented Information Logistics (i.e., the delivery of information at the right time, place, and with the right quality and format to the user) advocates data movement (D'Andria et al., 2015). The issue of inter-process communication has been faced also in Vitali and Pernici (2016) based on healthcare scenario. In this work, hidden dependencies between the processes of different organizations where discovered by taking advantage of the data collected by the IoT devices in the environment. By combining and analyzing the information generated by different actors, an improved coordination between stakeholders can thus be reached. The issue of how to collect and manage these data remains open.

The approach proposed in this paper to express requirements about data movement relies on goal-based models that are usually adopted in requirement engineering to specify the objectives of users and applications to be designed (Van Lamsweerde, 2001; Amyot and Mussbacher, 2011; Horkoff et al., 2015). By using the tree-like structures of goal models, decisions on which subset of the modeled goals must be achieved can be taken. To this aim, several techniques have been proposed (Letier and Van Lamsweerde, 2004; Horkoff and Yu, 2016). The satisfaction analyzes propagate the satisfaction or denial of goals forward and backward in the goal tree structure. The forward propagation (Letier and Van Lamsweerde, 2004) can be used to check alternatives, while the backward propagation (Giorgini et al., 2003; Sebastiani et al., 2004; Chung et al., 2012) can be used to understand what are the consequences of a satisfied or denied goal.

Among the several requirements that can be expressed through our application of the goal-based model, the quality of data and quality of service aspects are the most relevant ones; in this paper, they are considered together under the data utility umbrella. Data utility has been defined in different ways in the literature. In statistics (i), it has been defined as “A summary term describing the value of a given data release as an analytical resource. This comprises the data's analytical completeness and its analytical validity” (Hundepool et al., 2012). In business (ii), it has been defined as “business value attributed to data within specific usage contexts” (Syed et al., 2008). In IT environments (iii), it has been defined as as “The relevance of a piece of information to the context it refers to and how much it differs from other similar pieces of information and contributes to reduce uncertainty” (Kock and Kock, 2007). More related to a Fog computing environment, (Cappiello et al., 2017) defines data utility as a numeric measure that reflects the relative importance and value contribution of a record from a business/usage perspective and provides a flexible approach that has been adopted in this paper to cover different types of applications as well as customizable set of data quality parameters. In fact, in the literature, some papers consider data utility in specific usage contexts (Ives et al., 1983; Lin et al., 2015; Wang et al., 2016) or on a specific set of data quality dimensions, e.g., accuracy, accessibility, completeness, currency, reliability, timeliness, and usability (Skyrme, 1994; Moody and Walsh, 1999).

## 9. Concluding Remarks

The ever-growing adoption of IoT-based solutions in the healthcare sector has resulted in a significant increase in data production, which could have the potential to be used in internal hospital processes but could also be relevant externally. On this basis, this document presented an approach based on the Fog computing paradigm, which demonstrates how this paradigm fits perfectly as a way to organize a distributed software solution in which data is produced at the edge of the network and consumed in other nodes that could be internal or external while preserving the data utility requirements. This goal was achieved by considering a formalization of data utility defined as a combination of data quality and quality of service. In addition, a goal-based model approach is adopted to select and enact an adaptation action capable of recovering the situation in the event that data utility is not satisfied.

## Data Availability Statement

The datasets generated for this study are available on request to the corresponding author.

## Ethics Statement

The studies involving human participants were reviewed and approved by Ospedale San Raffaele Ethical Committee (Protocollo ISEE - 720571-1 Title: Use of sensorized smartglasses for user's context and activity awareness: a validation study). The patients/participants provided their written informed consent to participate in this study.

## Author Contributions

All authors listed have made a substantial, direct and intellectual contribution to the work, and approved it for publication.

## Conflict of Interest

The authors declare that the research was conducted in the absence of any commercial or financial relationships that could be construed as a potential conflict of interest.

## Footnotes

^1^http://www.ditas-project.eu

^2^The influence of the network could be impactful to the point of making the data movement via network intractable. For this reason, Amazon offers a service called Snowball (https://aws.amazon.com/snowball/) to securely and efficiently move huge amount of data by physically moving the data storage devices.

^3^https://isee-project.eu/

^4^The following notation style is adopted: variables are strings in italic with a leading non-capital letter (e.g., *x, y*); sets are strings with the leading letter in the calligraphic font for mathematical expressions (e.g., G, I).

^5^For the sake of clarity and without losing generality, in this paper we assume in the following examples to have a Fog environment composed of only two layers, i.e., the edge and the cloud. When considering more layers, the formalization of the data movement slightly changes by introducing an index, which reflects the position on the hierarchy where the lower values represent the edge and the higher ones the cloud.

^6^https://github.com/DITAS-Project/decision-system-for-data-and-computation-movement/
